# In vitro study of dentinal tubule penetration and filling quality of bioceramic sealer

**DOI:** 10.1371/journal.pone.0192248

**Published:** 2018-02-01

**Authors:** Yahui Wang, Siyi Liu, Yanmei Dong

**Affiliations:** Department of Cariology and Endodontology, Peking University School and Hospital of Stomatology, Beijing, P.R.China; Virginia Commonwealth University, UNITED STATES

## Abstract

This study aimed to evaluate the dentinal tubule penetration and filling quality of a bioceramic sealer (iRoot SP). Forty-two roots of extracted adult lower incisors were selected. After instrumentation with Protaper Universal to F3, 40 roots were chosen and randomly divided into 4 groups, as follows: iRoot SP single cone group, iRoot SP warm vertical group, AH Plus single cone group, and AH Plus warm vertical group. Before root canal filling, sealers were mixed with Rhodamine B dye for visualization under confocal laser scanning microscope. All samples were sectioned at 2, 4, and 6 mm to apex. Then, the percentages of void areas, gap regions, and segments of sealer that penetrated into dentinal tubules in each section were calculated. Non-parametric test was used for statistical analysis (α = 0.05). We found that filling techniques and types of sealer had no statistically significant effects on the occurrence of voids and gaps. The segments of iRoot SP penetrated into dentinal tubules were statistically more than that of AH Plus in both single cone and warm vertical techniques at 2 mm to apex (*P* < 0.05). Regardless of the filling technique used, iRoot SP can achieve comparable filling quality and better dentinal tubules penetration than AH Plus. Considering the good bioactivity of iRoot SP, it may help improve the seal of root canal system.

## Introduction

The complete sealing and filling of the cleaned and shaped root canal system are important steps that can affect the long term success of root canal treatment [1, 2]. Because of the complexity of root canal system, sealers need to be used to fill the irregularities and to penetrate into dentinal tubules to obtain a hermetic seal of the root canal system. Meanwhile, root canal sealers should provide adherence between gutta-percha and dentinal walls to avoid gap occurrence at the sealer-dentine interface[3].

Grossman[4] outlined the properties of an ideal sealer, including the following: provides good adhesion between it and the canal wall when set; establishes a hermetic seal; no shrinkage upon setting; insoluble in tissue fluids; tissue tolerant; and others. Current available commercial sealers can be broadly categorized into the following groups: zinc oxide eugenol-based, calcium hydroxide-based, glass ionomer-based, resin-based, silicone-based, and the recently introduced, calcium silicate based sealers. However, at present not one of the existing sealers satisfies all the criteria. Zinc oxide eugenol-based[5], calcium hydroxide-based[6] and glass ionomer-based sealers have the common problem of dissolving when in contact with periradicular tissues. In addition, zinc oxide eugenol-based sealer shrinks slightly when setting [7, 8]. AH Plus is an epoxy resin-based sealer with good physicochemical properties[9] and antibacterial effect[10]. It is a commonly used sealer in clinical practice. If extruded into the periapical tissues, AH Plus does not resorb easily [11] and can produce a short-term inflammatory response. AH Plus exhibits slight shrinkage after being immersed in water for 30 days, even if it meets the criteria of ISO 6876/2001[12].

In recent years, calcium silicate based materials have attracted considerable attentions because of their good biocompatibility and bioactivity. According to its manufacturers, iRoot SP (Innovative BioCreamix Inc., Vancouver, Canada), also called Endosequence BC Sealer (Brasseler, Savannah, GA), is a premixed, ready-to-use, and injectable bioceramic sealer. It is composed of calcium silicates, calcium phosphate monobasic, calcium hydroxide, zirconium oxide, filler, and thickening agents. Many in vitro studies have indicated its good biocompatibility [13–16], bioactivity [17, 18], antibacterial property [19–21], and certain kinds of sealing ability [22, 23]. Moreover, it has favorable flowability, small particle size, no setting shrinkage, and shows some extent of volume expansion [12, 24], which directly affect the root canal filling quality.

Single cone technique is a filling technique that uses a single matched gutta-percha cone and sealers. It is simple, easy to master, and saves time for clinicians. Neither longitudinal nor lateral pressure existing in condensed filling techniques [25, 26] is used on the root canal walls in single cone technique. Therefore, the risk of root fracture is decreased in teeth filled by using the single cone technique. In addition, no thermal damage to periodontal membrane is expected when the single cone technique is used, although this might occur when warm vertical technique is used [27]. However, because no condensation pressure exists during the filling procedure, the canal always contains a mass of sealers, which is much more than that obturated by cold lateral and warm vertical techniques [28]. The shrinkage during setting and dissolution when contacted with tissue fluid are common problems in most of the currently available sealers[5]. These shortcomings will result in the appearance of gaps in the interface of sealers and canal wall and microleakage in the root canal system after some time; such gaps ultimately affect the outcome of root canal treatment. As a result, single cone technique has rarely been used in the past years.

With its properties of no shrinkage when setting and no dissolution when in contact with tissue fluids, the new bioceramic sealer may be a promising filling material to overcome these issues. However, studies on the dentinal tubules penetration and filling quality of iRoot SP are limited and lead to inconsistent results. Akcay et al.[29] tested dentinal tubule penetration of AH Plus, iRoot SP, MTA Fillapex, and GuttaFlow Bioseal root canal sealers with single cone technique after different final irrigation procedures. They found that iRoot SP exhibited a significantly higher penetration area than the other groups. Fernandez et al.[30]found that iRoot SP with continuous wave of condensation was more effective in the filling of artificial lateral canals than the single-point technique. Celikten et al.[31]used Micro-CT in vitro to evaluate root canal sealer filling quality using a single-cone technique in oval-shaped canals and suggested a decrease in void formation of iRoot SP in the apical third. Some researchers[32] observed that iRoot SP showed more gaps compared with AH Plus when canals were filled via the cold lateral technique. This study aimed to evaluate the effects of the bioceramic sealer iRoot SP on root canal filling density, root canal adaptation, and sealer penetration into dentinal tubules when both single cone and warm vertical techniques were used.

## Materials and methods

### Sample selection and root canal instrumentation

This study was approved by the Ethics Committee, School and Hospital of Stomatology, Peking University (PKUSSIRB-201631102) and abides by the Declaration of Helsinki (version VI, 2002). After informing and obtaining the patients’ verbal consent, a total of 42 adult mandibular incisors with single straight canal extracted because of periodontitis were included. These specimens were stored in saline solution and then refrigerated at 4°C after scaling the calculi and periodontal membranes. The crowns were removed at the cementoenamel junction using a 0.3 mm low-speed diamond saw (SYJ-150, Shenyang Kejing Auto-instrument Co., Ltd., Shenyang, China). The working length was determined by subtracting 1 mm from the length of a size 10 K-file (Dentsply Maillefer) until it reached the apical foramen under stereomicroscope at 10×. Root canal was instrumented using rotary Ni-Ti instruments ProTaper Universal (Dentsply Maillefer) at the working length until the F3 (30, 0.09 taper) instrument. After each instrument was used, the canals were irrigated using 2 mL 2.5% sodium hypochlorite. Then, passive ultrasonic irrigation with 2.5% sodium hypochlorite was performed, as described by van der Sluis et al.[33]. A flush of 2 mL 17% EDTA was applied for 3 min to eliminate the smear layer. Finally, the canals were washed with 2 mL distilled water and dried with paper points.

### Root canal obturation

Two samples with no fillings were selected as blank control, and the rest of the specimens were randomly divided into four experiment groups according to sealers and filling techniques used (each group, n = 10), as follows:

*Group 1*: AH Plus with single cone technique (AH-SC group)
*Group 2*: iRoot SP with single cone technique (SP-SC group)
*Group 3*: AH Plus with warm vertical technique (AH-WV group)
*Group 4*: iRoot SP with warm vertical technique (SP-WV group)

AH Plus (Dentsply International, York, PA) was manipulated with 1:1 proportion according to the manufacturer’s instructions. To allow visualization under a confocal laser scanning microscope (CLSM, LSM 5 Exciter, Zeiss, Germany), the sealers were mixed with fluorescent Rhodamine B dye (Sigma-Aldrich, St Louis, MO) until an approximate concentration of 0.1 mg/ml was reached. All root canals were smeared with the Rhodamine B labeled sealers using a matched F3 gutta-percha cone (Dentsply Maillefer). Then, the canals were filled with SC or WV technique. The coronal opening was sealed with a temporary filling material (Ceivitron, Taiwan Dongquan Int Co., Ltd., Taiwan).

Finally, the specimens were stored at 37°C and 100% relative humidity for 1 week to allow complete setting of the sealers.

### Specimen analysis

After the setting of the root canal sealer, each specimen was embedded using cold self-curing resin and then sectioned perpendicular to its long axis using a 0.3 mm low-speed saw at 200 rpm with continuous water cooling. The horizontal level was set at 2, 4, and 6 mm to apex. The coronal section was observed under stereomicroscope (M125, Leica, Germany) and CLSM, as described by Moon et al.[34]. To visualize and obtain accurate images, we observed and recorded the pictures at 10μm below the surface using the 5× lens with a size of 1024 × 1024 pixels. The excitation and emission wavelengths for Rhodamine B were set at 543 and 590 nm respectively. Image J1.46r software was used to measure the area and perimeter of each root canal on the section. Afterward, the length of the root canal with gaps region and the segment with sealer penetration into dentinal tubules were determined. The percentages of gap regions and segment of sealer penetration into dentinal tubules were calculated.

The images of each specimen under stereomicroscope were observed and recorded at 80× magnification. Only sections without crack or with crack but no Rhodamine B filtration were used as proper samples. Red color appeared if cracks were dyed with Rhodamine B. Image J1.46r software was used to measure the void area and the area of each root canal section. Then, we obtained the value of the percentage of void areas on each root section.

### Statistical analysis

Because of the absence of normal distribution, statistical analysis was performed by using the nonparametric Kruskal-Wallis tests within groups (*P* < 0.05). The nonparametric Mann-Whitney test was used to analyze the differences between sealers and between filling techniques (*P* < 0.05). Data statistical analysis was conducted by using SPSS 16.0 software.

## Results

**Fig 1:**
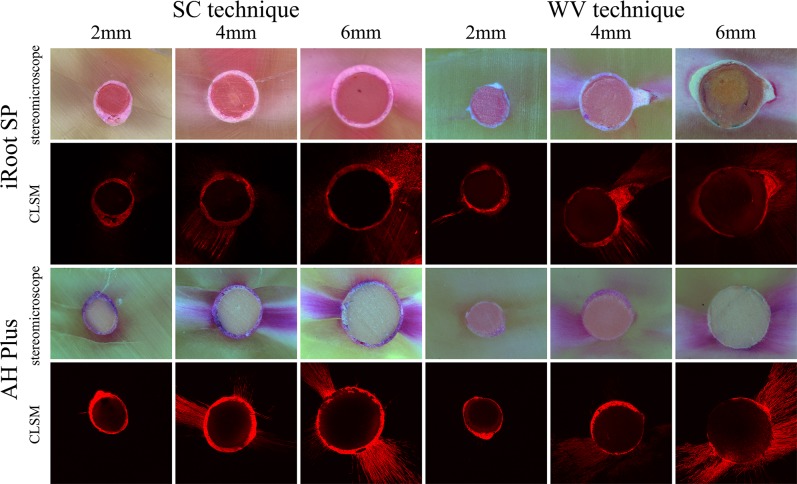
The representative images of stereomicroscope and confocal laser scanning microscope (CLSM) of root sections at 2, 4, and 6 mm levels to apex after obturated using iRoot SP or AH Plus with single cone (SC) and warm vertical technique (WV), respectively.

The typical images under stereomicroscope (80×) and CLSM (50×) were showed in Fig 1.

### The density of root canal filling

**Fig 2:**
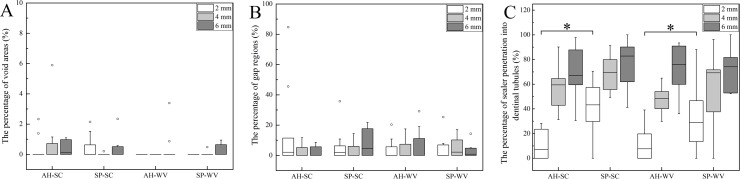
Box plot of percentage of void areas, gap regions and sealer penetration into dentinal tubules. (A) Box plot of percentage of void areas at 2, 4, and 6 mm to the apex. (B) Box plot of percentage of gap regions at 2, 4, and 6 mm to the apex. (C) Box plot of percentage of sealer penetration into dentinal tubules at 2, 4, and 6 mm to the apex. *: P < 0.05.

The medians of percentages of void areas of AH-SC, SP-SC, AH-WV, and SP-WV these four groups at 2, 4, and 6 mm level mostly were 0.00%, and no statistically significant difference was observed, with *P* = 0.117, 0.063, and 0.624, respectively (Figs 1 and 2A). In the SP-WV group, more void areas were observed at 6 mm level than that at 2 mm level, *P* = 0.031. Root canal filling techniques and types of sealer had no significant effect on the occurrence of voids.

### The adaptation to root canal wall

The medians of percentages of gap regions of AH-SC, SP-SC, AH-WV, and SP-WV at 2, 4 and 6 mm levels ranged from 0.00% to 4.42%, and no statistically significant differences were obtained, at *P* = 0.658, 0.740, 0.724, respectively. There was no statistical difference at 2, 4, and 6 mm among these three levels in each group (*P* > 0.05, Figs 1 and 2B). Root canal filling techniques and types of sealer had no significant influence on the adaptation to root canal wall.

### The penetration into dentinal tubules

(1) The medians of the percentages of penetrated segment of root canal at 2, 4, and 6 mm levels were 7.03%–43.20%, 48.34%–69.26%, and 66.98%–82.81%, respectively. No matter which kind of sealers or filling techniques was used, the penetrated segment of root canal increased from apical to coronal part of root. (2) More penetrated segments of the root canal were observed in the horizontal section at 2 mm level in groups SP-SC and SP-WV than in groups AH-SC (*P* = 0.002) and AH-WV (*P* = 0.033). iRoot SP was able to penetrate into and seal more dentinal tubules at 2 mm level than AH Plus. There were no statistical differences between groups AH-SC and AH-WV and between groups SP-SC and SP-WV. (3) At the horizontal levels of 4 and 6mm, there were no statistically significant differences in the penetrated segment of root canal between these four groups, with *P* = 0.085, 0.092, respectively (Figs 1 and 2C).

## Discussion

Currently, root canal sealer penetration into dentinal tubules was mainly tested by scanning electron microscopy (SEM) and CLSM. Methods to evaluate root canal filling quality include root sections followed by stereomicroscope, SEM, CLSM, and micro computerized tomography (micro CT). This study observed root sections after root canal filling by using CLSM to estimate the penetration ability and interfacial adaptation of a new bioceramic sealer and by using stereomicroscope to evaluate the filling density. The CLSM was used to evaluate the penetration ability of iRoot SP because it could provide a detailed view of the presence and distribution of sealers inside dentinal tubules when fluorescent Rhodamine B was added into the sealers. Another advantage of using CLSM is that the samples can be visualized in various depths, and it can differentiate the genuine interfacial failures from artificial gaps that could be produced after high vacuum desiccation under a scanning electron microscope [35, 36]. In addition, CLSM does not require any special specimen processing, and the observations can be made under close-to-normal conditions[3]. Micro CT is a non-destructive analytical method that provides 3D objective data with high accuracy and spatial resolution [31]. The micro CT was popularly used to estimate the root canal filling quality and to detect dentine defects such as cracks at pre- and post-root canal instrumentation and obturation [37–41]. A recent research [42] compared the filling quality of a calcium silicate-based sealer (Endoseal MTA) with single cone technique (EMS group) and AH Plus with warm vertical technique using both micro CT and root sections by stereomicroscope. No significant difference was found among groups evaluated by micro CT, whereas in the stereomicroscopic evaluation, the EMS group showed a higher number of voids and a higher void score compared with the other groups. Hence, they speculated that micro CT observations might be less sensitive compared with the sectioning method in terms of void detection. They suggested that the microscopic observations of the specimen obtained by sectioning should be included as a supportive method for evaluating the quality of root canal filling. With regards to dentine crack, we selected root sections with no crack or with crack but no Rhodamine B filtration by using stereomicroscope at 80× magnification. Another reason for not using micro CT to detect dentine crack was based on the evidence of a recent study[41], which used micro CT and root sections with stereomicroscope simultaneously to evaluate the occurrence of dentine crack. Unexpectedly, the images of stereomicroscope after root canal instrumentation exhibited the dentine crack, but the same image did not appeared on the micro CT images showing the pre- and post-instrumentation of the same section. The authors indicated that the formation of new cracks resulted from the sectioning procedure itself and not because of the mechanized instrumentation. Therefore, from this perspective, the Rhodamine B in this study was able to represent the sealers penetration into dentinal tubules.

As a kind of epoxy resin-based sealer, AH Plus is used frequently in clinical work and is usually chosen as the control in studies on the properties of new sealers because of its good flowability, proper film thickness, and viscosity [12]. Thus, AH Plus was also chosen as a control in this study.

In this study, only the percentage of penetrated segment of root canal was used to assess the penetration of iRoot SP, while previous studies [35, 43] also used maximum penetration depth as the evaluating indicator. However, on the one hand, penetration of sealers into dentinal tubules can form a physical barrier to prevent bacterial microleakage and recontamination of root canal system[44]. On the other hand, sealers penetrated into dentinal tubules can maintain their bactericidal effect [45, 46], which is favorable for the healing of the periapical lesion. Therefore, compared with the maximum penetration depth, the percentage of penetration segment probably had more meaning and clinical relevance [47].

Regarding the penetration property of sealers, we found out that iRoot SP penetrated more segments of root canal at 2 mm level than AH Plus, when both single cone and warm vertical techniques were used. This probably is related to its high flowability and smaller particle size [12, 24]. Similarly, Akcay et al. [29] tested the dentinal tubule penetration of AH Plus, iRoot SP, MTA Fillapex, and GuttaFlow Bioseal root canal sealers with single cone technique after different final irrigation procedures. iRoot SP group exhibited a significantly higher penetration area than the other groups. In the present study, root canal filling techniques had no effect on the penetrated segment of the root canal, although a significant reduction of the flow of iRoot SP was found at high temperature [48]. We speculated that there were two reasons for this phenomenon, as follows. (1) Heat conducted a limited distance in root canal so that the apical part of root canal filled by warm vertical technique was actually filled by using the single cone technique. (2) With increasing number and diameter of dentinal tubules, neglecting the influence of filling technique on the penetrated segment of root canal became possible. Consistent with previous studies[36, 43], we observed that regardless of the kind of sealers or filling techniques used, the percentages of penetrated segment of root canal increased from apical to coronal part. This was due to the increase in number and diameter of dentinal tubules[49] and the increase of elimination of smear layer in the upper middle section of the root canal.

A study[50] indicated that sealer penetration into dentinal tubules had no correlation with the sealability of nonbonded root fillings, but it was actually of paramount clinical relevance, particularly for iRoot SP. The iRoot SP penetration into dentinal tubules will generate micromechanical interlocking with root dentine and strengthen the resistance of the filling material. In addition, the moisture remaining in the dentinal tubules will trigger its setting reaction with the production of hydroxyapatite, thereby creating the chemical bond with root dentine [17]. The micromechanical interlocking along with the chemical bond between iRoot SP and root dentine improve resistance to filling material dislocation and probably strengthen the root to prevent fracture[51].

In our study, regardless of whether single cone technique or warm vertical technique was used, no significant differences in the occurrence of voids and gaps in root canal were found. iRoot SP could have parallel filling quality with AH Plus when these two techniques were used. A recent study[52] evaluated the presence of voids after continuous wave of condensation and single-cone obturation with AH Plus sealer in mandibular molars using micro CT. No significant difference was found on the total percentage volume of voids between the two obturation techniques; only in the cervical third, voids volume in continuous wave of condensation was less than that in single cone obturation. More void areas were found at 6 mm level than that at 2 mm level in the SP-WV group, which was in consistent with Celikten’s study [31]. They used micro CT in vitro to evaluate root canal sealer filling quality using the single cone technique in oval shaped canals and suggested a decrease in the void formation of iRoot SP in the apical third; a significant difference was found between the apical and coronal thirds. However, during the procedure, we observed that iRoot SP became set, and smog appeared as soon as it came in contact with heat. Moreover, the extra gutta-percha could not be removed out of the canal easily, thereby influencing the handling characteristic. This might have been the reason for the appearance of more voids at 6 mm level than 2 mm level in the SP-WV group.

In this study, we newly found that bioceramic sealer iRoot SP penetrated into dentinal tubules well and better than AH Plus in the 2 mm to the apex. Apical third is the most complex and critical area in the root canal system, not only for root canal instrumentation but also for root canal filling[53]. As early as 1994, Oguntebi[54] reported that root canal infection and reinfection may occur following pulp necrosis or during and after endodontic treatment because of bacteria, which exist in dentinal tubules. He suggested that strategies designed to eliminate this microflora must include agents that can penetrate the dentinal tubules and destroy these microorganisms. The good penetration ability of iRoot SP may be one of factors responsible for the outcome of endodontic treatment; this requires demonstration in the future.

## Conclusion

With the limitations of this study, we draw the conclusion that the bioceramic sealer (iRoot SP) had sufficient filling quality and better dentinal tubules penetration regardless of the filling technique used. The better dentinal tubules penetration of iRoot SP combined with its good bioactivity may help improve the seal of root canal system. This conclusion needs further investigation.
